# Supplementary material for: Reg4 and complement factor D prevent the overgrowth of E. coli in the mouse gut
Source: Commun Biol. 2020 Sep 2;3:483. doi: 10.1038/s42003-020-01219-2 (PMC7468294; doi:10.1038/s42003-020-01219-2)
Supplement: Supplementary file 1 — Supplementary Information [file 42003_2020_1219_MOESM1_ESM.docx]

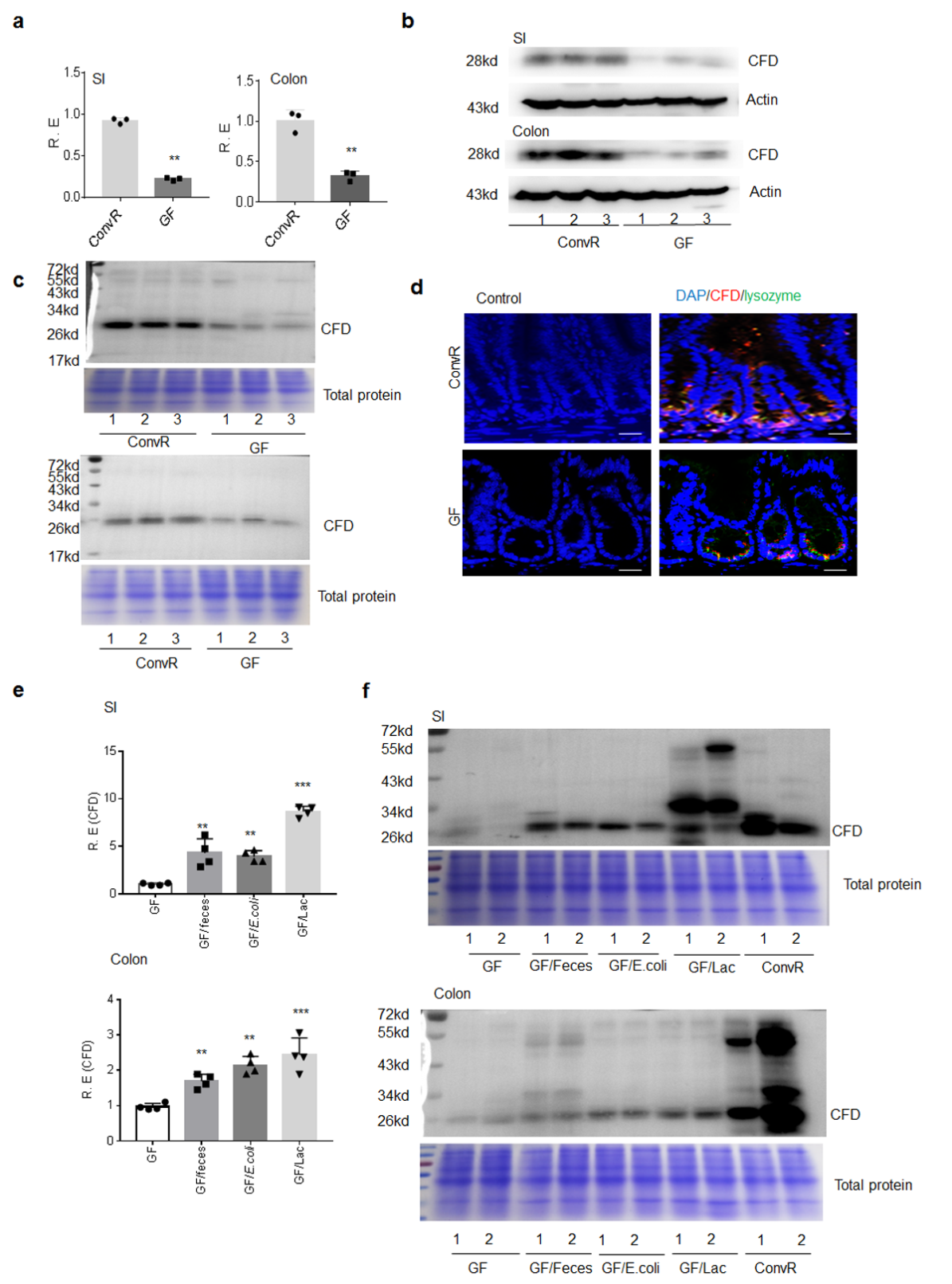


**Supplementary Fig. 1. Gut bacteria upregulate the expression CFD-related to main text Figure 1.**

**a** qRT-PCR of CFD in small intestine (SI) and colonic tissues of no DSS treated conventionally raised (ConvR) and germ-free (GF) mice (Pooled sample, n=3).

**b** Immunoblotting of CFD in small intestine (SI) and colon tissues of no DSS-treated ConvR and GF mice. Number, different individuals.

**c** Immunoblotting of CFD in equal amount of small intestine (SI) and colon contents of no DSS treated ConvR and GF mice. Total protein was stained using Coomassie. Number, different individuals.

**d** Immunostaining of CFD in colonic tissues of no DSS treated ConvR and GF mice. One representative of five mice.

**e** QRT-PCR of CFD in small intestine (SI) and colon tissues of GF mice after infusing ConvR mouse feces (GF/feces), *E. coli* 0160 (GF/E.coli), which are isolated from colitis tissues and *L. Reuteri* (GF/Lac). *Lactobacillus reuteri* (ATCC PTA 4659) is from BioGaaia, Sweden. GF, uninfused control GF mice.

**f** Immunoblotting of CFD in equal amount of small intestine (SI) and colon contents of GF mice after infusing ConvR mouse feces (GF/feces), E. coli 0160 (GF/E.coli) and *L. Reuteri* (GF/Lac). Total protein was stained using Coomassie. Number, different individuals.

Two side Student’s *t*-test in a; ANOVA plus post-Bonferroni analysis in e; *P<0.05, **P<0.01 and ***P<0.001; R. E, relative expression.

**
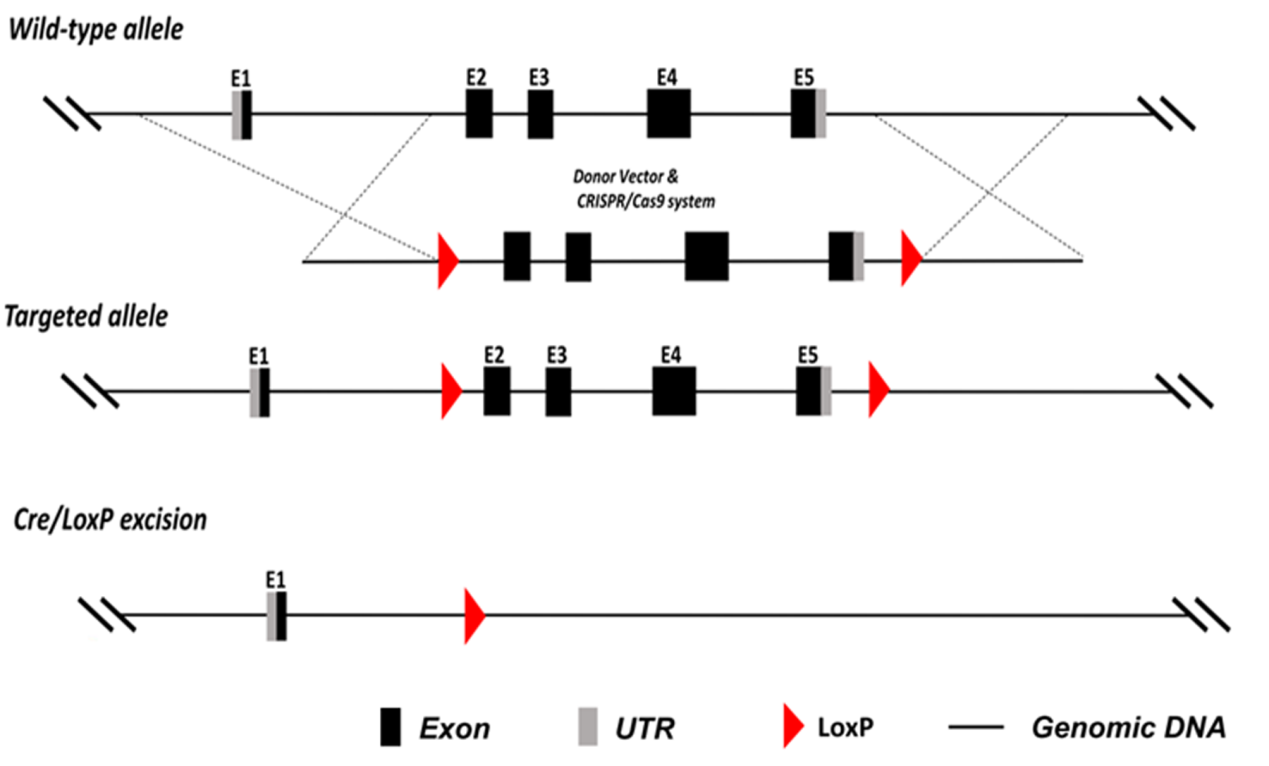
**

**Supplementary Fig. 2. Preparation of gut CFD conditional knockout mice -Related to main text Figure 1.**

**
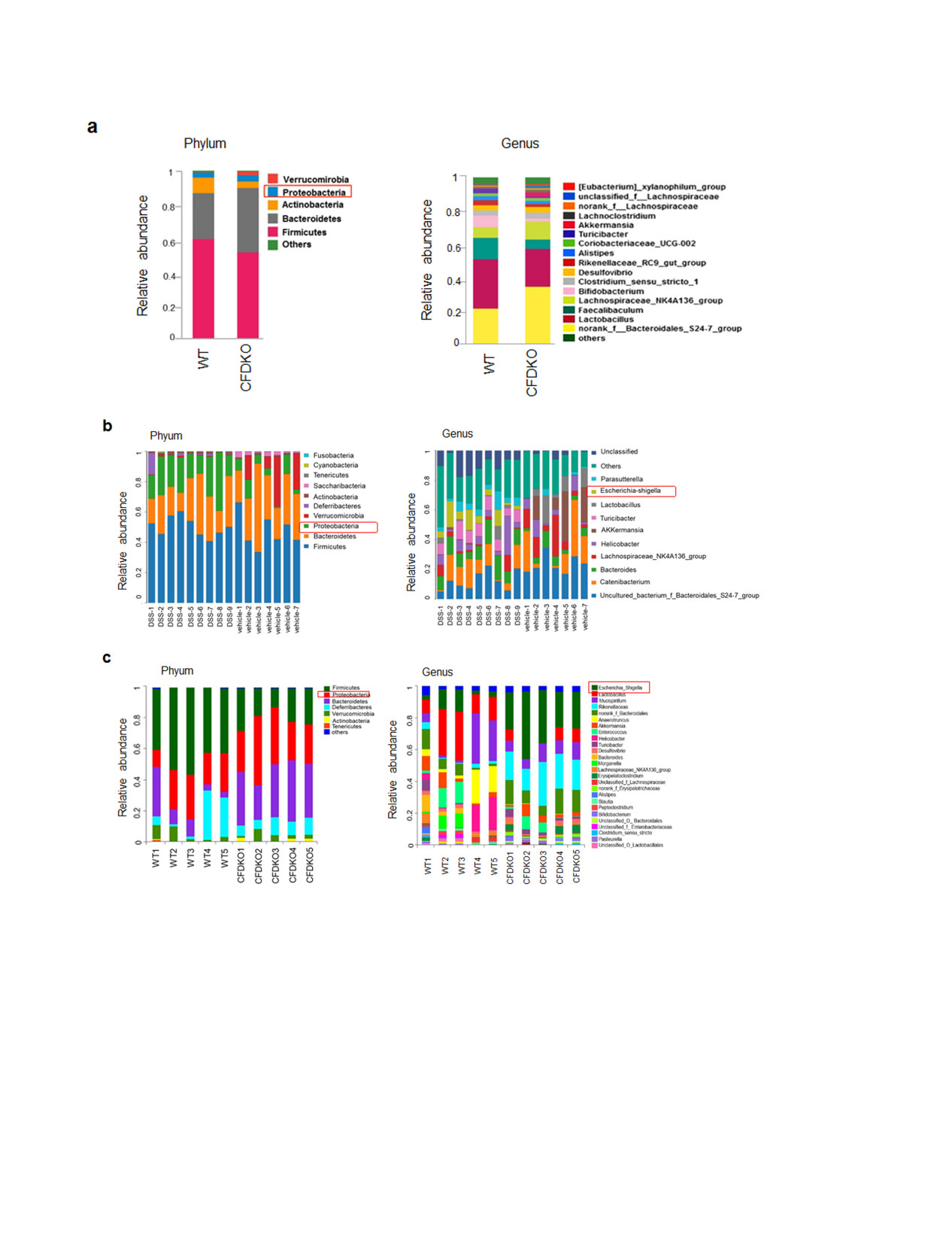
Supplementary Fig. 3. 16SrRNA analyses of gut contents in normal, DSS-treated WT and CFD KO mice-Related to main text Figure 2.**

**a** 16SrRNA analyses of colonic contents in normal CfD^fl/fl^pvillin-cre^T^ (CFDKO) mice and their cohoused CfD^fl/fl^pvillin-cre^w^ (WT) mice (Pooled sample, n=6). The samples were clustered at phylum or genus levels using the sample phylum or genus count matrices.

**b** 16SrRNA analyses of colonic contents in WT mice with or without 2 % DSS treatment. The samples were clustered at phylum or genus levels using the sample phylum or genus count matrices. DSS 1-9, mice were fed with a 2 % DSS solution in drinking water for 7 days; Vehicle 1-7, mice were only fed with drinking water.

**c** 16SrRNA analyses of colon contents in CFD^fl/fl^pvillin-cre^T^ (CFD) mice and their cohoused CFD^fl/fl^pvillin-cre^w^ (WT) mice after 2 % DSS treatment (n=5). The samples were clustered at phylum or genus levels using the sample phylum or genus count matrices.


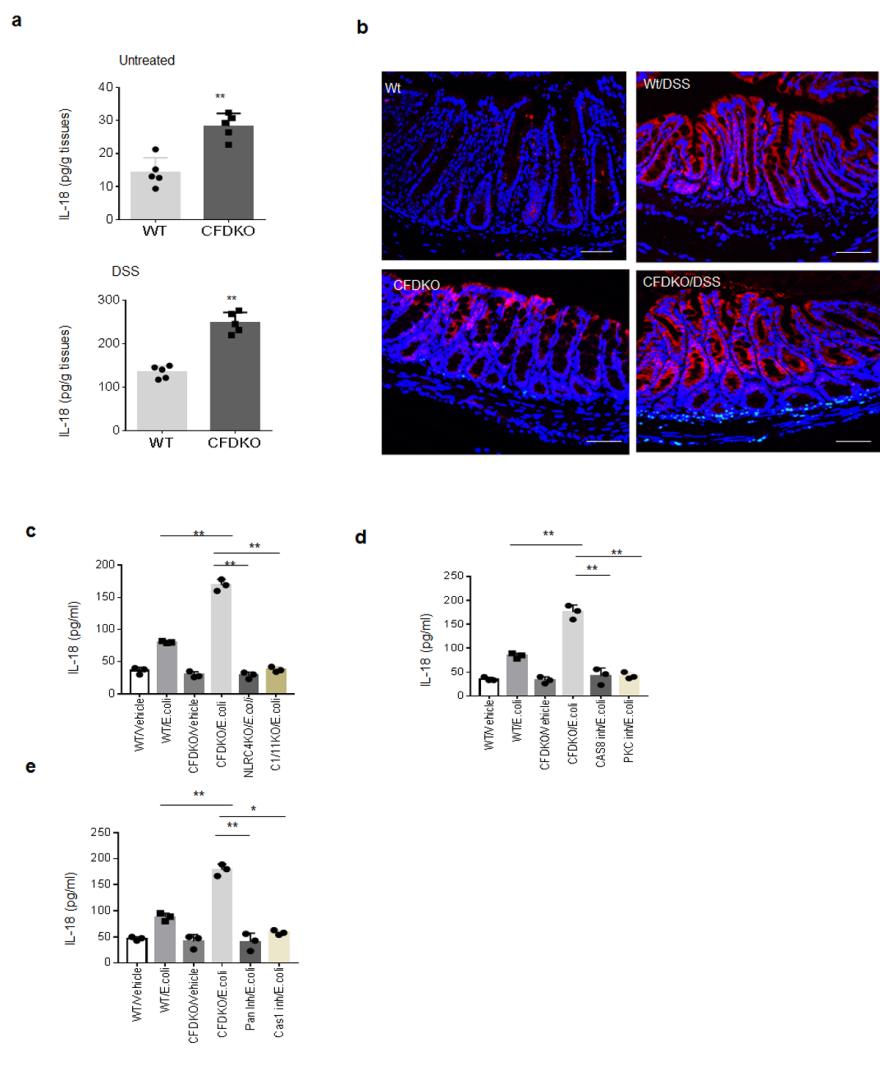


**Supplementary Fig. 4. *E.coli* 0160 isolated from DSS-treated mice induces expression of IL-18 in CfD^fl/fl^pvillin-cre^T^ mice-Related to main text Figure 3.**

**a** ELISA of IL-18 in colon tissues of CFD^fl/fl^pvillin-cre^w^ (WT) and CFD^fl/fl^pvillin-cre^T^ (CFDKO) mice with (DSS) or without (Untreated) DSS treatment.

**b** Immunostaining of IL-18 in colon tissues of CFD^fl/fl^pvillin-cre^w^ (WT) and CfD^fl/fl^pvillin-cre^T^ mice (CFDKO) with (WT/DSS or CFDKO/DSS) or without DSS treatment (WT or CFDKO).

**c** ELISA of mature IL-18 in colon tissues of WT (WT/E.coli), CFD^fl/fl^pvillin-cre^T^ (CFDKO/E.coli), NLRC4 KO (NLRC4 KO/E.coli) and CASPASE1/11 KO (caspase 1/11 KO/E.coli) after infusing *E. coli* 0160 (0160) (Pooled sample, n=3). WT/Vehicle and CFDKO/vehicle, controls.

**d** ELISA of mature IL-18 in colon tissues of CFD^fl/fl^pvillin-cre^T^ mice (CFDKO) with or without caspase 8 inhibitor (Cas8inh/E.coli) or PKCδ inhibitor (PKCinh/E.coli) after infusing *E. coli* 0160 (Pooled sample, n=3). WT and CFDKO/Vehicle, controls.

**e** ELISA of mature IL-18 in colon tissues of CFD^fl/fl^pvillin-cre^T^ mice (CFDKO) with or without pan-caspase inhibitor (Paninh/E.coli) or caspase 1 inhibitor (Cas1inh/E.coli) after infusing E. coli 0160 (0160, pooled sample, n=3). WT and CFDKO/Vehicle, controls.

Data in c, d and e were from tissues treated *ex vivo.*

Two side Student’s *t*-test in A; ANOVA plus post-Bonferroni analysis in c, d and e; *P<0.05, **P<0.01 and ***P<0.001; NS, no significance.

**
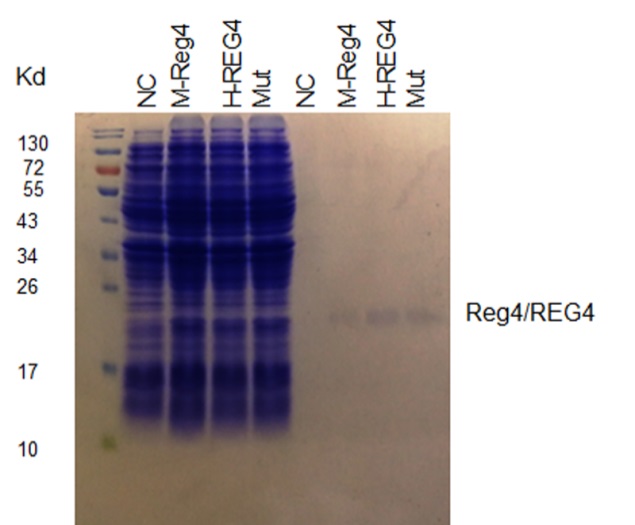
**

**Supplementary Fig. 5. Preparation of mouse Reg4, human REG4 and mutant human REG4 -Related to main text Figure 5.**

NC (negative control), M-Reg4 (mouse Reg4), H-REG4 (human REG4) and Mut (mutant human REG4) in left indicate input of different groups; whereas in right indicate proteins in different groups after purification.

**
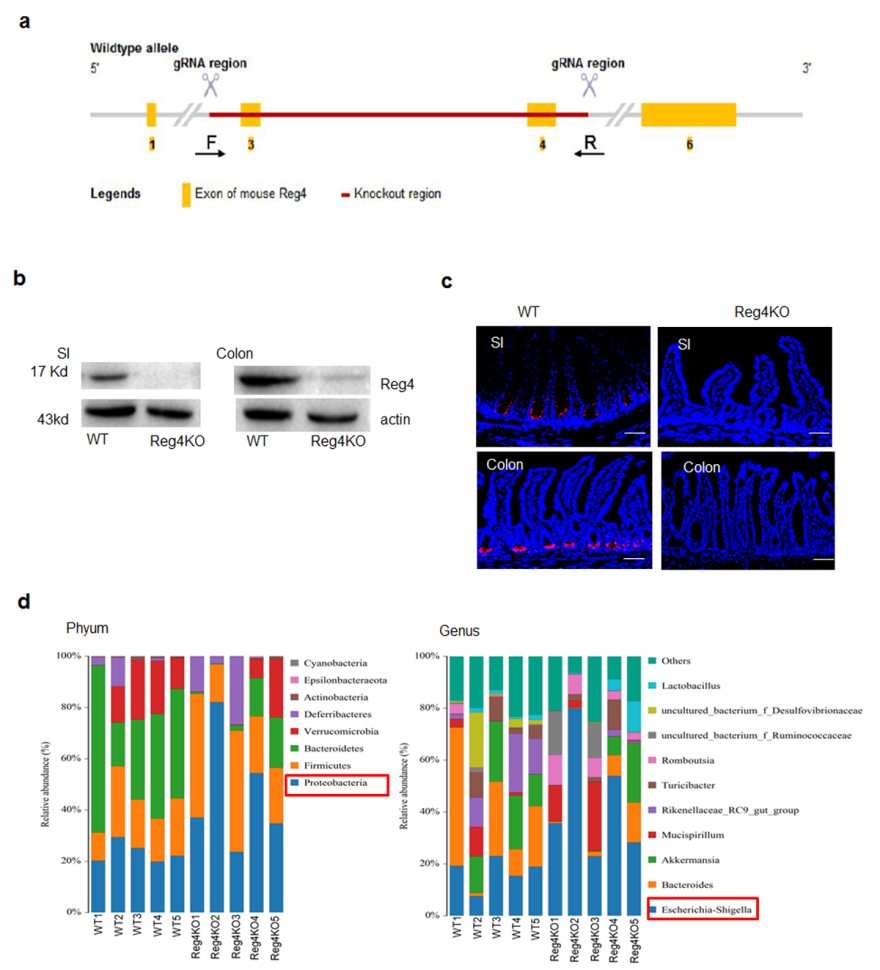
**

**Supplementary Fig. 6. 16S rRNA Analyses of Colon Contents in WT and REG4 KO Mice -Related to main text Figure 7**.

**a** Strategy for generating Reg4 KO mice.

**b** Immunoblotting of Reg4 in the small intestine (SI) and colonic tissues of WT and Reg4 KO mice.

**c** Immunostaining of Reg4 in small intestine (SI) and colon of WT and Reg4 KO mice.

**d** 16S rRNA analyses of colon contents of WT and Reg4 KO mice after 2% DSS treatment. The samples were clustered at phylum or genus levels using the sample phylum or genus count matrices.

**
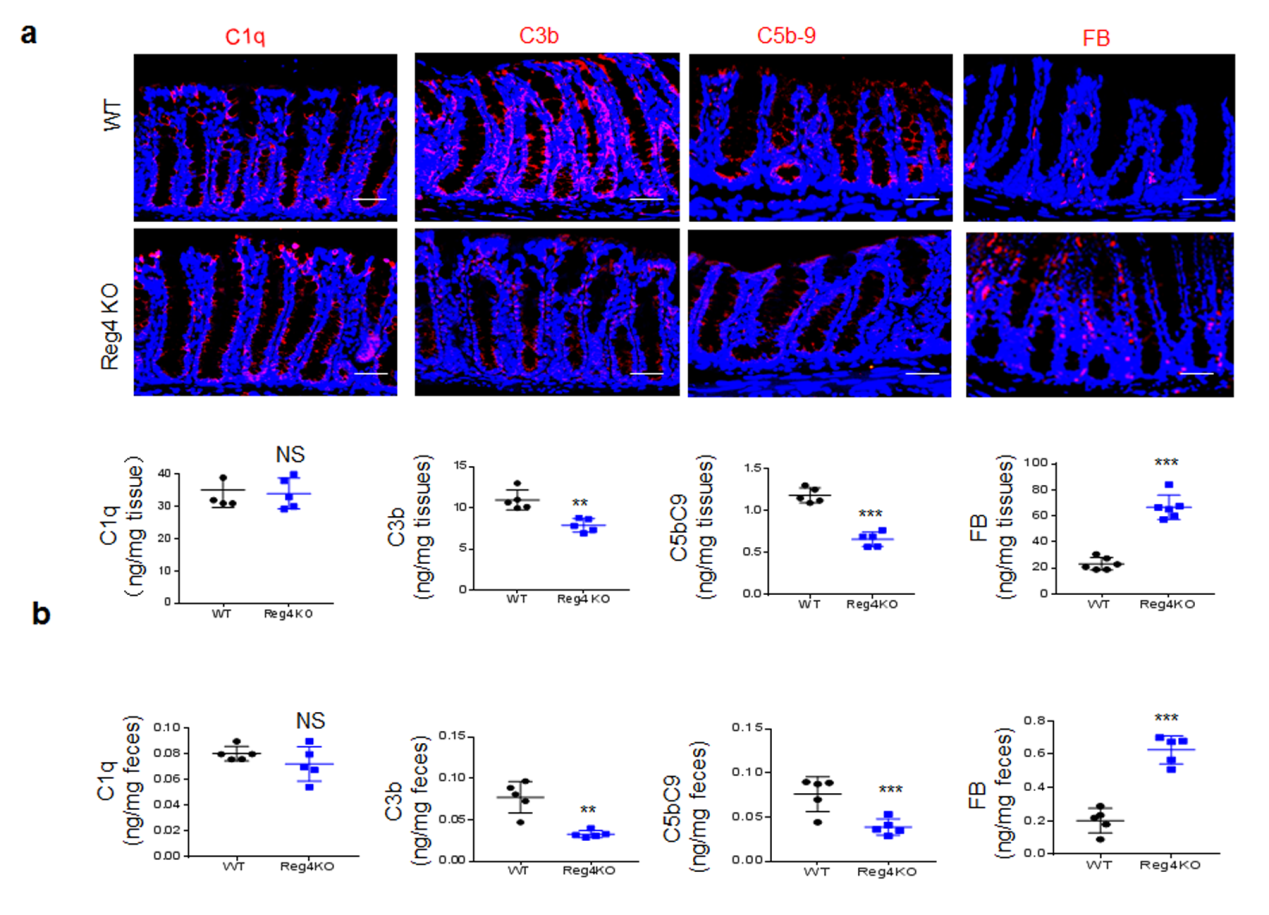
**

**Supplementary Fig. 7. Immunostaining and ELISA of C1q, C3, C5b-9 and FB in the colonic tissues and feces-Related to main text Figure 7.**

**a** Immunostaining (upper) and ELISA (lower) of C1q, C3, C5b-9 and FB in the colon tissues of WT and Reg4 KO mice (n=5).

**b** ELISA of C1q, C3, C5b-9 and FB in the feces of WT and Reg4 KO mice (n=5).

Two side Student’s *t*-test in a and b; *P<0.05, **P<0.01 and ***P<0.001; NS, no significance.

**
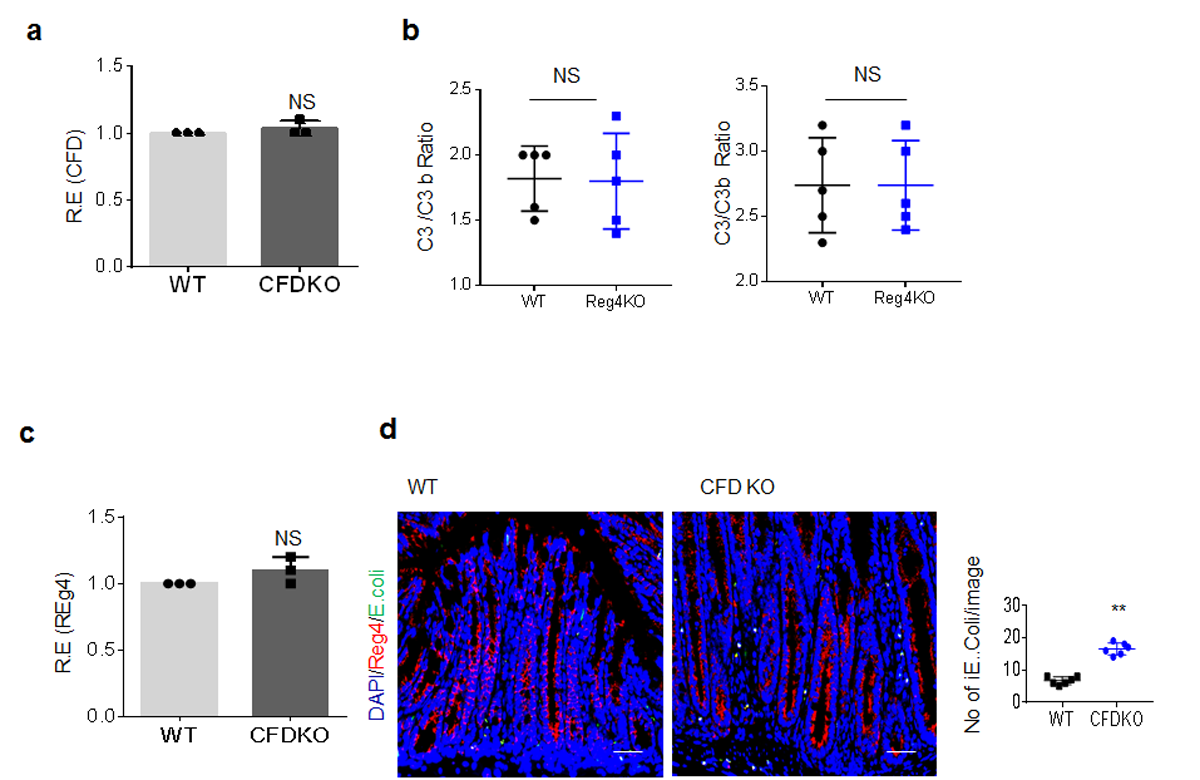
**

**Supplementary Fig. 8. Activity of CFD in Reg4 deficient mice and vice versa.** a qRT-PCR of CFD in the colon tissues of *wt* and *Reg4* KO mice; b C3/C3b ratio in colon tissues (left) and in feces (right). C3 and C3b were detected using Elisa, and then ratio of C3 and C3b was calculated. c qRT-PCR of Reg4 in the colon tissues of *wt* and *CFD* KO mice. d Immunostaining of Reg4 bound *E. coli* in colon tissues. Reg4 bound E. coli were compared between *wt* and *CFD* KO mice. “**”$<0.05; NS, no signi$ficance.

**
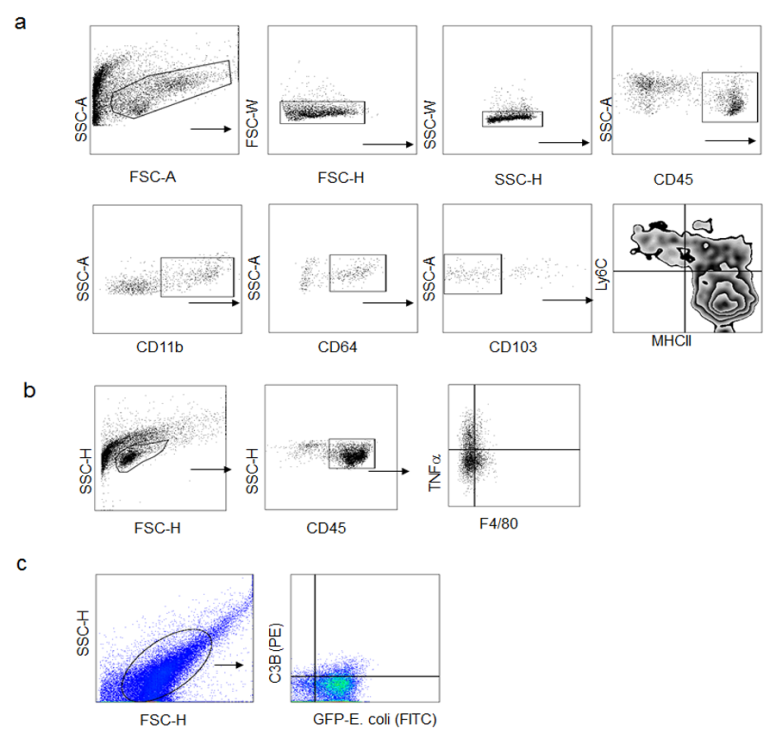
**

**Supplementary Fig. 9. Gated strategies for flow cytometry. a,** Figure 1i, 1 j and figure 3g; **b,** Figure 1i, 1 j and figure 3g; **c,** Figure 4g, figure 6b, and figure 7f.

**
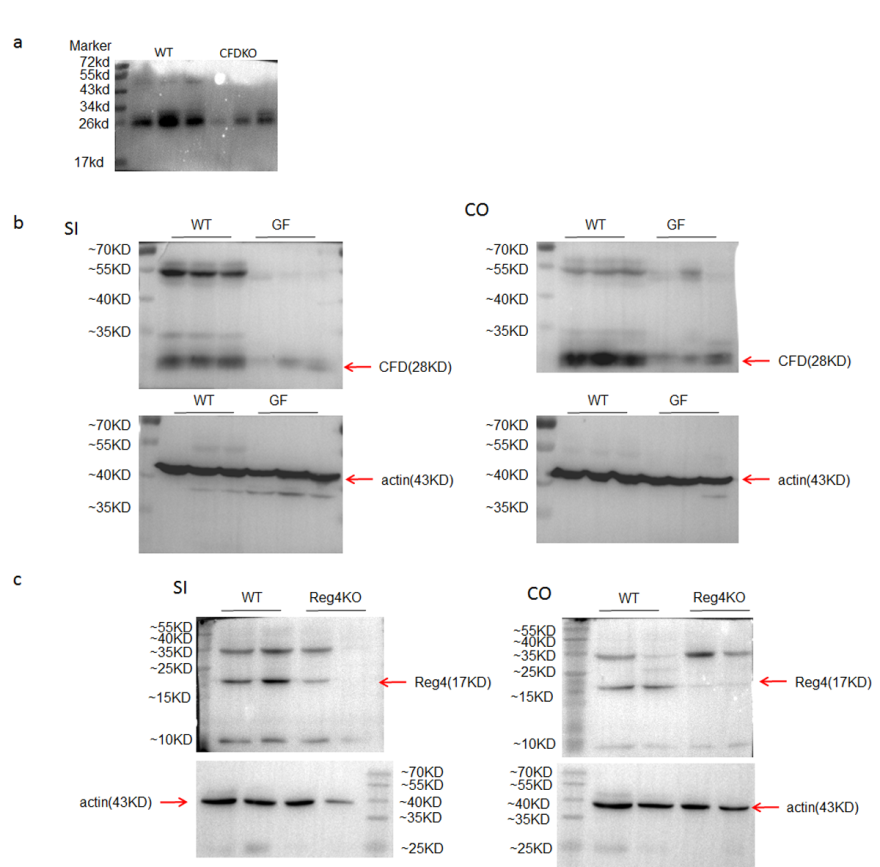
**

**Supplementary Fig. 10. The full, uncropped blot/gel images. a,** Figure 1a; **b,** Supplementary figure 1b**; C,** Supplementary figure 6b.
