# Supplementary material for: Reg4 and complement factor D prevent the overgrowth of E. coli in the mouse gut
Source: Commun Biol. 2020 Sep 2;3:483. doi: 10.1038/s42003-020-01219-2 (PMC7468294; doi:10.1038/s42003-020-01219-2)
Supplement: Supplementary file 2 — Description of Additional Supplementary Files [file 42003_2020_1219_MOESM2_ESM.docx]

**Description of Additional Supplementary Files：**

**File Name：Supplementary Data 1**

Description: Reagents and oligoes used in this study.

**File Name: Supplementary Data 2**

Description: Source data for the plots.
