# Supplementary material for: Reg4 and complement factor D prevent the overgrowth of E. coli in the mouse gut
Source: Commun Biol. 2020 Sep 2;3:483. doi: 10.1038/s42003-020-01219-2 (PMC7468294; doi:10.1038/s42003-020-01219-2)
Supplement: Supplementary file 3 — Supplementary Data 1 [file 42003_2020_1219_MOESM3_ESM.pdf]

Supplementary Table 1. Reagents and oligoes used in this study.

REAGENT or RESOURCE	SOURCE	IDENTIFIER
Antibodies for immunoblotting and immunostaining		
β-Actin (C4) mouse	Santa Cruz	Cat: sc-47778 RRID:AB_626632
FITC-Goat Anti-Rat IgG(H+L)	Proteintech	Cat: SA00003-11
Alexa Fluor 488-Goat Anti-Mouse IgG(H+L)	Proteintech	Cat: SA00006-1
Alexa Fluor 594-Goat Anti-Rabbit IgG(H+L)	Proteintech	Cat: SA00006-4
Alexa Fluor 488-Goat Anti-Rabbit IgG(H+L)	Proteintech	Cat: SA00006-2
Alexa Fluor 594-Goat Anti-Mouse IgG(H+L)	Proteintech	Cat: SA00006-3
FITC-Rabbit Anti-goat IgG(H+L)	Proteintech	Cat: SA00003-4
Anti-Mouse MUC2	Santa Cruz	Cat: sc-15334
Anti-Mouse F4/80	Santa Cruz	Cat: sc-71088
Anti-Mouse TNFa	Santa Cruz	Cat: sc-52746
Anti-Mouse C3b	Bioss	Cat: bs-4873R
Anti-Mouse IgA	Santa Cruz	Cat: sc-373823
Anti-Mouse Reg4	Santa Cruz	Cat: sc-67187
Anti-Mouse C1q	Bioss	Cat: bs-11337R
Anti-Mouse C5b-9	Bioss	Cat: bs-2673R
Anti-Mouse FB	Santa Cruz	Cat: sc-47681
Anti-Mouse Collectin	Proteintech	Cat: 15269-1-AP
Anti-Mouse CFD	Santa Cruz	Cat: sc-376015
Anti-Mouse MASP1/3	Santa Cruz	Cat: sc-166815
Anti-Mouse MASP2	Bioss	Cat: bs-1980R
Anti-Mouse CK19(A-3)	Santa Cruz Biotechnology	Cat:sc-376126 RRID:AB_10988034
Anti-Mouse CD11b (1B6e)	Santa Cruz Biotechnology	Cat: sc-21744 RRID:AB_626882
Anti-Mouse IL-18	Abcam	Cat: ab71495 RRID:AB_1209302
Anti-lysozyme	Santa Cruz	sc-27956
**Antibodies for flow cytometry**		
PerCP/Cy5.5-CD45 (30-F11) mouse	Biolegend	Cat:103132 RRID:AB_893340
FITC anti-mouse CD4 (RM4-5)	Thermo Fisher Scientific	Cat:11-0042-85 RRID:AB_464897
PE anti-mouse IFNgamma (XGM1.2)	Thermo Fisher	Cat:25-7311-82 RRID:AB_469680

	Scientific	
APC anti-mouse TNFa (MP6-XT22)	Thermo Fisher Scientific	Cat: 17-7321-82 RRID:AB_469508
Percp/cy5.5 anti-mouse NKp46(29A1.4)	Biolegend	Cat:137610 RRID:AB_10641137
PE anti-mouse IL10(JES5-16E3)	Thermo Fisher Scientific	Cat: 12-7101-82
APC-IL17 (eBio17B7) mouse	eBioscience	Cat:11-7177-81 RRID:AB_763581
FITC-F4/80 (BM8) mouse	Biolegend	Cat:123108 RRID:AB_893502
PE anti-mouse Foxp3 (NRRF-30)	Thermo Fisher Scientific	Cat: 12-4771-82 RRID:AB_529580
APC-CD11c (N418) mouse	Biolegend	Cat:117310 RRID:AB_313779
PE-CD103 (2E7) mouse	Biolegend	Cat:121405 RRID:AB_535948
PerCP/Cy5.5-CD11b (M1/70) mouse/human	Biolegend	Cat:101227 RRID:AB_893233
PE anti-mouse MHCII (M5/114.15.2)	Biolegend	Cat:107608 RRID:AB_313323
PE-Ly6G (1A8) mouse	BD Bioscience	Cat:551461 RRID:AB_394208
FITC-Ly6C (AL-21) mouse	BD Bioscience	Cat:553104 RRID:AB_394628
Anti-LPS	Abcam	Ab54089
Primers for Real-time PCR		
Murine GAPDH-Fs	BGI	5’-TCAACGGCACAGTCAAGG-3’
Murine GAPDH-Rs	BGI	5’-TACTCAGCACCGGCCTCA-3’
Murine TNFa-Fs	BGI	5’-GGTCTGGGCCATAGAACTGA-3’
Murine TNFa-Rs	BGI	5’-CAGCCTCTTCTCATTCCTGC-3’
Murine IL-6-Fs	BGI	5’-TCTGAAGGACTCTGGCTTTG-3’
Murine IL-6-Rs	BGI	5’-GATGGATGCTACCAAACTGGA-3’
Murine IL-1β-Fs	BGI	5’-GTGTCTTTCCCGTGGACCTT-3’
Murine IL-1β-Rs	BGI	5’-AATGGGAACGTCACACACCA-3’
Primers for detection of bacteria		
16s 27F	BGI	5’- AGAGTTTGATCCTGGCTCAG-3’
16s 1492R	BGI	5’- GGTTACCTTGTTACGACTT-3’
Eubacteria-Fs	BGI	5’- ACTCCTACGGGAGGCAGCAGT-3’
Eubacteria-Rs	BGI	5’-ATTACCGCGGCTGCTGGC-3’
E. coli-Fs	BGI	5’- TGGGATCTCCATTGTCAGA-3’
E. coli-Rs	BGI	5’-CACTGGTGTGGGCCATAATTC -3’
Bacteroides Phylum-Fs	BGI	5’-GAGAGGAAGGTCCCCCAC -3’
Bacteroides Phylum-Rs	BGI	5’-CGCTACTTGGCTGGTTCAG -3’
Firmicutes Phylum-Fs	BGI	5’-GCTGCTAATACCGCATGATATGTC -3’

Firmicutes Phylum-Rs	BGI	5’-CAGACGCGAGTCCATCTCAGA -3’
**Primers for generation and identifition of CFD mice**		
Vil1-ProF1	BGI	5’- GTGTTTGGTTTGGTTTCCTCTGCATAAGA-3’
Cre5R1	BGI	5’- GCAGGCAAATTTTGGTGTACGGTCA-3’
Adipsin-flox-TF	BGI	5’- TCCGTGTACTTCGTGGCTCT-3’
Adipsin-flox-TR	BGI	5’- TCTTGTTCATGGCCGCTCT-3’
Adipsin-1stloxp-tF	BGI	5’- CACAGCTCCGTGTACTTCGTG-3’
Adipsin-1stloxp-tR	BGI	5’- CTGAAGACAACTTGTAGCGAT-3’
Adipsin-2ndloxp-TF	BGI	5’- TGTATGCTATACGAAGTTATTGGGCTCAGACAG-3’
Adipsin-3rm-tR1	BGI	5’- AGTCAGACAGGGTAACAGTC-3’
Probe		
Ecoli	BGI	cy3-GAG ACT CAA GAT TGC CAG TAT CAG
**Critical Commercial Assays**		
Normal sera	Quidel	Cat: A112
C1 delete sera	Quidel	Cat: A509
C3 delete sera	Quidel	Cat: A508
CFD delete sera	Quidel	Cat: A525
Mouse IFNgamma (37895) Ab antibody	RD Systems	Cat: MAB485 RRID:AB_2123047
Mouse C1q ELISA Kit	Elabscience	Cat: LS-F8963
Mouse C3b ELISA Kit	Elabscience	Cat: E-EL-M0330c
Mouse C5b-9 ELISA Kit	Elabscience	Cat: E-EL-M1129c
Mouse FB ELISA Kit	Elabscience	Cat: E-EL-M0334c
Mouse IL-18 ELISA KIT	Elabscience	Cat: E-EL-M0730c
QIAquick PCR Purification Kit	Qiagen	Cat:28104
QuantiTect SYBR Green PCR Master Mix	Qiagen	Cat:208052
Foxp3 / Transcription Factor Fixation/Permeabilization Concentrate and Diluent	Thermo Fisher	Cat: 00-5521-00
Cell stimulation cocktail	ebioscience	Cat: 00-4975-03
Permeabilization Buffer	Thermo Fisher	Cat: 00-8333-56
ECL chemiluminescence	Absin	Cat: abs920
Protease Inhibitor Cocktail	Sigma-Aldrich	Cat: P8340
**Other reagents**		
DSS	Mpbio	Cat: 0216011080
Ampicillin	Sigma	Cat: BP021

Vancomycine	Sigma	Cat: V2002
Neomycin sulfate	Sigma	Cat: N6386
Metronidazole	Sigma	Cat: M3761
MacConkey	Solarbio	Cat: LA8200
MacConkey Agar	Solarbio	Cat: M8560
Trizol	Life Technologies	Cat: 15596026
FBS	Gibco	Cat:10099141
Collagenase IV	Sigma	Cat: C5138
Dnase I	Solarbio	Cat: D8071
DMEM	Gibco	Cat:11965118
HBSS	Gibco	Cat:14170161
Pecoll	Solarbio	Cat: P8370
PMA	Sigma	Cat: 79346
GolgiStop	BD Biosciences	Cat: 554724
Peptidoglycan(PGN)	Sigma-Aldrich	Cat: 72789
Lipopolysaccharides(LPS)	Sigma-Aldrich	Cat: L2630
Mannan	Sigma-Aldrich	M7504
IgA	Sigma-Aldrich	Cat: I4036
Pan caspase inhibitor (Z-VAD-FMK)	ApexBio Technology	Cat: A1902
Caspase1 inhibitor (Z-YVAD-FMK)	ApexBio Technology	Cat: A8955
Caspase8 inhibitor(Z-IETD-FMK)	ApexBio Technology	Cat: B3232
PKCδ inhibitor (Rottlerin)	MedChemExpress	Cat: HY-18980
