# Supplementary material for: Reg4 and complement factor D prevent the overgrowth of E. coli in the mouse gut
Source: Commun Biol. 2020 Sep 2;3:483. doi: 10.1038/s42003-020-01219-2 (PMC7468294; doi:10.1038/s42003-020-01219-2)
Supplement: Supplementary file 5 — Reporting Summary [file 42003_2020_1219_MOESM5_ESM.pdf]

nature research

Corresponding author(s): Rongcun Yang

Last updated by author(s): Jul 28, 2020

Reporting Summary

Nature Research wishes to improve the reproducibility of the work that we publish. This form provides structure for consistency and transparency in reporting. For further information on Nature Research policies, see Authors & Referees and the Editorial Policy Checklist.

Statistics

For all statistical analyses, confirm that the following items are present in the figure legend, table legend, main text, or Methods section.

n/a	Confirmed	
☐	☒	The exact sample size (n) for each experimental group/condition, given as a discrete number and unit of measurement
☐	☒	A statement on whether measurements were taken from distinct samples or whether the same sample was measured repeatedly
☐	☒	The statistical test(s) used AND whether they are one- or two-sided<br>*Only common tests should be described solely by name; describe more complex techniques in the Methods section.*
☐	☒	A description of all covariates tested
☐	☒	A description of any assumptions or corrections, such as tests of normality and adjustment for multiple comparisons
☐	☒	A full description of the statistical parameters including central tendency (e.g. means) or other basic estimates (e.g. regression coefficient) AND variation (e.g. standard deviation) or associated estimates of uncertainty (e.g. confidence intervals)
☐	☒	For null hypothesis testing, the test statistic (e.g. F, t, r) with confidence intervals, effect sizes, degrees of freedom and P value noted<br>*Give P values as exact values whenever suitable.*
☒	☐	For Bayesian analysis, information on the choice of priors and Markov chain Monte Carlo settings
☒	☐	For hierarchical and complex designs, identification of the appropriate level for tests and full reporting of outcomes
☒	☐	Estimates of effect sizes (e.g. Cohen's d, Pearson's r), indicating how they were calculated

Our web collection on statistics for biologists contains articles on many of the points above.

Software and code

Policy information about availability of computer code

Data collection	No software was used
Data analysis	Student's t-test and ONE-way ANOVA Bonferroni's Multiple Comparison Test was used to determine significance. The statistical significance of the survival curves was estimated using Kaplan and Meier method, and the curves were compared using the generalized Wilcoxon's test. Histological scores in different groups were analyzed by a Mann-Whitney U test. A 95% confidence interval was considered significant and was defined as $p < 0.05$. * indicates $p < 0.05$, ** $p < 0.01$, *** $p < 0.001$.

For manuscripts utilizing custom algorithms or software that are central to the research but not yet described in published literature, software must be made available to editors/reviewers. We strongly encourage code deposition in a community repository (e.g. GitHub). See the Nature Research guidelines for submitting code & software for further information.

Data

Policy information about availability of data

All manuscripts must include a data availability statement. This statement should provide the following information, where applicable:

- Accession codes, unique identifiers, or web links for publicly available datasets
- A list of figures that have associated raw data
- A description of any restrictions on data availability

Sequencing data have been deposited. Source data are shown in Supplementary Data 2. All other data (if any) are available upon reasonable request.

Field-specific reporting

Please select the one below that is the best fit for your research. If you are not sure, read the appropriate sections before making your selection.

☒ Life sciences ☐ Behavioural & social sciences ☐ Ecological, evolutionary & environmental sciences

For a reference copy of the document with all sections, see nature.com/documents/nr-reporting-summary-flat.pdf

Life sciences study design

All studies must disclose on these points even when the disclosure is negative.

Sample size	n= >6
Data exclusions	N/A
Replication	For any experiment, will be repeated at least three times.
Randomization	N/A
Blinding	N/A

Reporting for specific materials, systems and methods

We require information from authors about some types of materials, experimental systems and methods used in many studies. Here, indicate whether each material, system or method listed is relevant to your study. If you are not sure if a list item applies to your research, read the appropriate section before selecting a response.

Materials & experimental systems

n/a	Involved in the study	
☐	☒	Antibodies
☒	☐	Eukaryotic cell lines
☒	☐	Palaeontology
☐	☒	Animals and other organisms
☒	☐	Human research participants
☒	☐	Clinical data

Methods

n/a	Involved in the study	
☒	☐	ChIP-seq
☐	☒	Flow cytometry
☒	☐	MRI-based neuroimaging

Antibodies

Antibodies used

Antibodies for immunoblotting and immunostaining

β-Actin (C4) mouse Santa Cruz Cat: sc-47778 RRID:AB_626632

FITC-Goat Anti-Rat IgG(H+L) Proteintech Cat: SA00003-11

Alexa Fluor 488-Goat Anti-Mouse IgG(H+L) Proteintech Cat: SA00006-1

Alexa Fluor 594-Goat Anti-Rabbit IgG(H+L) Proteintech Cat: SA00006-4

Alexa Fluor 488-Goat Anti-Rabbit IgG(H+L) Proteintech Cat: SA00006-2

Alexa Fluor 594-Goat Anti-Mouse IgG(H+L) Proteintech Cat: SA00006-3

FITC-Rabbit Anti-goat IgG(H+L) Proteintech Cat: SA00003-4

Anti-Mouse MUC2 Santa Cruz Cat: sc-15334

Anti-Mouse F4/80 Santa Cruz Cat: sc-71088

Anti-Mouse TNFa Santa Cruz Cat: sc-52746

Anti-Mouse C3b Bioss Cat: bs-4873R

Anti-Mouse IgA Santa Cruz Cat: sc-373823

Anti-Mouse Reg4 Santa Cruz Cat: sc-67187

Anti-Mouse C1q Bioss Cat: bs-11337R

Anti-Mouse C5b-9 Bioss Cat: bs-2673R

Anti-Mouse FB Santa Cruz Cat: sc-47681

Anti-Mouse Collectin Proteintech Cat: 15269-1-AP

Anti-Mouse CFD Santa Cruz Cat: sc-376015

Anti-Mouse MASP1/3 Santa Cruz Cat: sc-166815

Anti-Mouse MASP2 Bioss Cat: bs-1980R

Anti-Mouse CK19(A-3) Santa Cruz Biotechnology Cat:sc-376126 RRID:AB_10988034

Anti-Mouse CD11b (1B6e) Santa Cruz Biotechnology Cat: sc-21744 RRID:AB_626882

Anti-Mouse IL-18 Abcam Cat: ab71495 RRID:AB_1209302

Anti-lysozyme Santa Cruz sc-27956

Antibodies for flow cytometry

PerCP/Cy5.5-CD45 (30-F11) mouse Biolegend Cat:103132 RRID:AB_893340

FITC anti-mouse CD4 (RM4-5) Thermo Fisher Scientific Cat:11-0042-85 RRID:AB_464897

PE anti-mouse IFNgamma (XGM1.2) Thermo Fisher Scientific Cat:25-7311-82 RRID:AB_469680

APC anti-mouse TNFa (MP6-XT22) Thermo Fisher Scientific Cat: 17-7321-82 RRID:AB_469508

Percp/cy5.5 anti-mouse NKp46(29A1.4) Biolegend Cat:137610 RRID:AB_10641137

PE anti-mouse IL10(JES5-16E3) Thermo Fisher Scientific Cat: 12-7101-82

APC-IL17 (eBio17B7) mouse eBioscience Cat:11-7177-81 RRID:AB_763581

FITC-F4/80 (BM8) mouse Biolegend Cat:123108 RRID:AB_893502

PE anti-mouse Foxp3 (NRRF-30) Thermo Fisher Scientific Cat: 12-4771-82 RRID:AB_529580

APC-CD11c (N418) mouse Biolegend Cat:117310 RRID:AB_313779

PE-CD103 (2E7) mouse Biolegend Cat:121405 RRID:AB_535948

PerCP/Cy5.5-CD11b (M1/70) mouse/human Biolegend Cat:101227 RRID:AB_893233

PE anti-mouse MHCII (M5/114.15.2) Biolegend Cat:107608 RRID:AB_313323

PE-Ly6G (1A8) mouse BD Bioscience Cat:551461 RRID:AB_394208

FITC-Ly6C (AL-21) mouse BD Bioscience Cat:553104 RRID:AB_394628

Validation

All antibodies are validation for the species and application

Animals and other organisms

Policy information about studies involving animals; ARRIVE guidelines recommended for reporting animal research

Laboratory animals

Four-to six-week-old male or female C57BL/6 mice were obtained from Nanjing Animal Center. Caspase-1/Caspase 11-/-, and NLRC4-/- in B6 background from Prof. Meng in University of Chinese Academy of Sciences, shanghai and Prof. Shao in National Institute of Biological Sciences, Beijing were bred and kept under specific pathogen-free (SPF) condition in Nankai University.
C57BL/6 germ-free (GF) mice were generated by Beijing Animal Center.
CFDfl/flpvillin-creTmice (gut CFD conditional knockout mice) were prepared by NBRI, China;
Reg4 KO mice were generated by CYAGEN, China.

Wild animals

N/A

Field-collected samples | N/A

Ethics oversight | Animal experiments were approved by the Institute's Animal Ethics Committee of Nankai University.

Note that full information on the approval of the study protocol must also be provided in the manuscript.

Flow Cytometry

Plots

Confirm that:

☒ The axis labels state the marker and fluorochrome used (e.g. CD4-FITC).

☒ The axis scales are clearly visible. Include numbers along axes only for bottom left plot of group (a 'group' is an analysis of identical markers).

☒ All plots are contour plots with outliers or pseudocolor plots.

☒ A numerical value for number of cells or percentage (with statistics) is provided.

Methodology

Sample preparation

For the staining of lamina propria (LP) lymphocytes, colon or small intestine were isolated, cleaned by shaking in ice-cold PBS four times before tissue was cut into 1 cm pieces. The epithelial cells were removed by incubating the tissue in HBSS with 2 mM EDTA for 30 min at 37oC with shaking. The LP cells were isolated by incubating the tissues in digestion buffer (DMEM, 5% fetal bovine serum, 1 mg/ ml Collagenase IV (Sigma-Aldrich) and DNase I (Sigma-Aldrich) for 40 min. The digested tissues were then filtered through a 40-mm filter. Cells were resuspended in 10 ml of the 40% fraction of a 40: 80 Percoll gradient and overlaid on 5 ml of the 80% fraction in a 15 ml Falcon tube. Percoll gradient separation was performed by centrifugation for 20 min at 1,800 rpm at room temperature. LP cells were collected at the interphase of the Percoll gradient, washed and resuspended in medium, and then stained and analyzed by flow cytometry. Dead cells were eliminated through 7-AAD staining.
For analysis of different immune cell populations, the cells were washed with staining buffer containing 2% FBS, 1 mM EDTA and 0.09% NaN3 and surface staining was performed with APC, FITC, PercP, BV 605 or PE-labeled anti-CD4, CD8, NKp46, CD11c, MHCII, F4/80, CD11b, Ly6C and CD45 antibodies and analyzed using FACScan flow cytometry HYPERLINK \l "_ENREF_58" \o "Su, 2014 #302" 58.
For intracellular staining, the cells were cultured and stimulated for 6 hrs with 50ng/ml phorbol 12-myristate 13-acetate (PMA, Sigma) and 1μg/ml ionomycin (Sigma) in the presence of GolgiStop (10 ng/ml, BD Biosciences). After incubation for 6 hrs, cells were washed in PBS, and then fixed in Cytofix/Cytoperm, permeabilized with Perm/Wash buffer (BD Biosciences), and stained with FITC-, PE-, APC- APC/cy7-, PerCP/Cy5.5- or PE/cy7-conjugated antibodies. Meanwhile, dead cells were eliminated through 7-AAD staining.
For analyses of C3b-, Reg4-, IgA- or collectin-coating of E. coli in colonic contents, fresh colonic contents was resuspended in sterile PBS. An aliquot estimated to contain no more that GFP-labeled 106 E. coli was directly stained with a monoclonal body. After washing, secondary antibodies were added. After a final washing step, samples were analyzed on the BD flow cytometry with setting adapted for optimal detection of bacterial-sized particle.

Instrument | BD flow cytometry

Software | N/A

Cell population abundance | N/A

Gating strategy | Gating strategy was performed based on FSC/SSC, and then specific cell populations

☒ Tick this box to confirm that a figure exemplifying the gating strategy is provided in the Supplementary Information.
